# MISHIMA - a new method for high speed multiple alignment of nucleotide sequences of bacterial genome scale data

**DOI:** 10.1186/1471-2105-11-142

**Published:** 2010-03-18

**Authors:** Kirill Kryukov, Naruya Saitou

**Affiliations:** 1 Division of Population Genetics, National Institute of Genetics 1111 Yata, Mishima, 411-8540, Japan; 2 Genome Network Project, National Institute of Genetics 1111 Yata, Mishima, 411-8540, Japan

## Abstract

**Background:**

Large nucleotide sequence datasets are becoming increasingly common objects of comparison. Complete bacterial genomes are reported almost everyday. This creates challenges for developing new multiple sequence alignment methods. Conventional multiple alignment methods are based on pairwise alignment and/or progressive alignment techniques. These approaches have performance problems when the number of sequences is large and when dealing with genome scale sequences.

**Results:**

We present a new method of multiple sequence alignment, called MISHIMA (Method for Inferring Sequence History In terms of Multiple Alignment), that does not depend on pairwise sequence comparison. A new algorithm is used to quickly find rare oligonucleotide sequences shared by all sequences. Divide and conquer approach is then applied to break the sequences into fragments that can be aligned independently by an external alignment program. These partial alignments are assembled together to form a complete alignment of the original sequences.

**Conclusions:**

MISHIMA provides improved performance compared to the commonly used multiple alignment methods. As an example, six complete genome sequences of bacteria species *Helicobacter pylori *(about 1.7 Mb each) were successfully aligned in about 6 hours using a single PC.

## Background

Alignment of vast number of nucleotide sequences is one of the central problems in current genomic studies. Sequence alignment is used in many kinds of biological studies, including phylogenetic analysis, identification of conserved domains, prediction of protein structure and database searches.

Alignment of multiple sequences remains a considerable challenge in terms of computation time required to complete an alignment of a reasonably large sequence dataset. Even pairwise sequence alignment becomes hard when the sequences are very long. Most alignment algorithms use some sorts of heuristics to reduce the computation time. Every alignment method was designed with some expectations on the size of data to be analyzed and what sort of computer hardware is likely to be used. The progress in alignment techniques during the last 30 years would not be possible without the parallel constant improvement in computer technology. The alignment methods being developed these days work better than those of 20 ago partly just because they are designed to run on faster processors and use more memory.

The most simple and widely used such heuristic is the method of progressive alignment [1], which was employed in a number of alignment programs such as MULTALIGN [2], MULTAL [3], MultAlin [4], CLUSTAL W [5,6], and MUSCLE [7]. Under the idea of progressive alignment, first the sequences are compared pairwise, one to one. Each pair of sequences is aligned with the dynamic programming algorithm, and the evolutionary distance between the sequences is estimated. Based on those distances the phylogenetic tree is constructed and then used for building multiple alignment.

An alternative approach to solve the problem of multiple sequence alignment is the iterative procedure of refining the global alignment, for example in PRRP program [8]. Also a genetic algorithm for multiple sequence alignment was proposed [9]. MAFFT package [10] contains both progressive and iterative alignment methods, depending on a novel approach of finding homologous regions with the help of fast Fourier transform.

Another direction focuses on increasing the performance of traditional dynamic programming based methods. This includes Divide-and-Conquer techniques, implemented in DCA [11] and OMA [12]. There are also studies attempting to take advantage of the parallel computation, especially popular for progressive alignment techniques like CLUSTAL W [13-15].

Recently it became popular to locate regions of local conservations before attempting to construct the global alignment. This idea was used for pairwise alignment in MUMmer [16], Shuffle-LAGAN [17], Avid [18] and GS-Aligner [19]. For multiple sequences a similar technique was used in DIALIGN [20], CHAOS [21], and MAFFT [10].

While there is considerable progress in recent multiple alignment research, it still remains a challenging problem to align multiple genomic sequences. Therefore we investigated an non-conventional heuristic approach to multiple sequence analysis, which we present in this work. A prototype of MISHIMA was briefly introduced by Kryukov and Saitou [22].

## Implementation

### Extraction of important information

It is believed that only a certain part of a genome is functional, and most of the genome content is never expressed or used. As a result some parts of the genome remain mostly unchanged during the evolution, while other parts quickly accumulate mutations through neutral evolution [23]. Conserved parts of the genome can be very helpful in identifying homology. If we could quickly locate conserved areas in the genome, that information obtained could be used to assist the process of alignment.

This idea has already been implemented in other methods, in which homology was first detected by pairwise sequence comparison and the obtained information was then used to build the alignment (DIALIGN [20], MLAGAN [24]). However for large genomic sequences the pairwise comparison step is very time consuming. Also pairwise comparison does not reveal features shared by multiple sequences. On the other hand, it is often the case that several sequences share some regions of high similarity. We propose a new heuristic method for locating conserved patterns shared by multiple sequences that can be directly used in a sequence alignment procedure.

### Divide-and-Conquer approach

Rearrangements of DNA fragments are known to happen very rarely in the evolutionary process, once sequences started to evolve independently in different species. Majority of the mutations result from substitutions or insertion/deletion events. Therefore in most cases it is a valid approach to use divide-and-conquer procedure for sequence alignment. Its principle is that sequences can be split into parts that will be aligned independently and a complete alignment will finally be constructed by assembling the partial alignments together [11,25].

The main difficulty in this method is to find the splitting points. The DCA program uses a pairwise sequence comparison with a dynamic programming-based method to find the splitting positions [25]. However the cost of pairwise comparison increases rapidly with the increase of sequence length and/or number of sequences. Below we propose a more rapid method to find the splitting positions.

Once the sequences are divided into sufficiently short segments, these segments can be aligned by calling a conventional alignment program. DCA uses MSA alignment program [26,27] to align the parts of sequences. In this study we use CLUSTAL W [6] and MAFFT [10] to align the sequence segments separately.

CLUSTAL W is not suitable for aligning long sequences or large number of sequences because of high computational requirements. MAFFT can be very fast with small datasets, but its computation time increases rapidly when aligning larger datasets, like complete bacterial genomes. Application of these programs can be extended to much larger datasets by combining with the Divide-and-Conquer approach.

It should be noted that such Divide-and-Conquer approach is not able to detect duplications, inversions and genomic rearrangements. This approach is valid under the assumption that all sequences can be aligned in a linear fashion to each other to form a multiple alignment.

### Using k-tuple frequencies

The core idea of MISHIMA is to analyze k-tuples found in the original sequences and to evaluate them based on their frequencies. When a particular nucleotide k-tuple is found exactly once in each sequence, we consider it as a likely homology signal. A k-tuple that has its close variants found once in each sequence is considered to be less likely, but still possible homology signal. Analyzing all k-tuples up to certain length allows us to select those k-tuples that represent the most probable homology shared by multiple sequences. These k-tuples can then be used to anchor the sequences before employing Divide and Conquer method to complete the alignment.

The basic principle of MISHIMA can be outlined as follows:

1. Find potentially useful k-tuples based on the number of their occurrences in the sequence data.

2. Analyze the potentially useful k-tuples, and select those that represent most probable local homology.

3. Use the selected k-tuples as anchors, split the sequences into segments.

4. Align the segments independently from each other.

5. Join partial alignments to complete the final multiple sequence alignment.

### Dictionary of k-tuples

As a first step we count the number of occurrences of each short k-tuple in the original sequence dataset. We keep this information in a dictionary structure, indexed by a k-tuple sequence. The number of possible nucleotide k-tuples is 4^K^, so the maximum k we can use is limited by the amount of memory we can use for the dictionary. We store k-tuple frequencies as 32-bit numbers, so 4*4^K ^bytes is required to store the frequencies of all nucleotide k-tuples. This allowed us to use k of 13 and 14 on 32-bit machines.

We found that knowing the number of occurrences of each k-tuple in the original sequence dataset is not enough to efficiently decide which k-tuples are more likely to represent the local homology. Therefore we decided to also store the number of sequences exhibiting each k-tuple. Additionally we store the index of the last sequence where the k-tuple was found, which allows us to collect all the frequencies using single read through the sequence dataset.

### Choosing the right lengths of a k-tuple

Length k is a very important factor affecting the chance for a k-tuple to be useful for alignment purposes. A random sequence of length L is expected to contain L/4^K ^occurrences of each k-tuple. Let us take L as A*N, where N is the number of sequences and A is the average sequence length. The expected number of occurrences for one k-tuple is A*N/4^K^. Since we are mostly interested in finding k-tuples with exactly N occurrences (once in each sequence), an ideal case for this method would be where A*N/4^K ^= N, which means A = 4^K^. Thus, to have the best results with this method, the length of sequences to be analyzed should be comparable to, or shorter than 4^K^.

This is an intuitive estimation, to give some feeling of relation between the dataset size and k-tuple's k. Of course the real biological sequences, especially those that we are trying to align, are often not completely random, which suggests that longer sequences can be aligned if they contain significant homology. Therefore the capability of MISHIMA to align the real sequences should be higher than this estimate. But non-conserved intergenic regions in a genomic sequences may well be considered as random sequence, and the total size of such regions in a sequence data is a limiting factor for MISHIMA. This shows that the length k is the basic parameter affecting the performance of this method.

Choosing the optimal length of a k-tuple is critical for the successful alignment. If k is too small, too many occurrences of each k-tuple will be found. Although to some extent this depends on the length of the sequences, it is difficult to predict whether the particular k is too small or not. Therefore we use all k-tuples with k from 1 to M in this method. For selecting M - the upper limit of k - we suggest to depend on the memory limitation. Most of the steps of the MISHIMA algorithm are performed in linear time, which makes it practical to use the largest M allowed by the amount of available memory.

We ended up using 12 bytes for each k-tuple in a dictionary. The total amount of memory required for dictionary is therefore 12*(4+4^2^+...+4^M^) bytes. This allows us to use M of 12, with the dictionary occupying less than 300 MB of RAM.

### Finding seeds

Our initial method of using identical k-tuples shared by all sequences has a low sensitivity, and we decided to use inexact matching instead. We do this by updating the dictionary to include the number of inexact copies of a k-tuple in the sequence dataset. Our method allows up to one substitution difference between two k-tuples.

After a k-tuple dictionary is constructed and updated with inexact match counts, it becomes possible to select k-tuples which represent potential homology. Potentially useful k-tuples are defined as those that have one inexact match in every sequence. Such k-tuples, or seeds, are extracted and saved for further analysis.

The next step is to find the locations of seeds in sequence data. Locations are extracted in a second read through the sequence data. It should be noted that this step also completes in linear time.

In cases where no seeds could be found, the external aligner is used to align the whole dataset.

### Seed compatibility

Now we will introduce measure for compatibility of two seeds, based on their relative coordinates in sequence data. For now we will consider only seeds that are found not more than once in every sequence. If both of two seeds, A and B, can be found in a sequence, there are two possible orders: either the coordinate of A is smaller than that of B, or vice versa. We can mark these two cases as A->B and B->A.

If all sequences in a dataset exhibit the same order of seeds A and B, these two seeds are compatible. However, if sequences have these seeds some in A->B order while some other in B->A order, we can then define an incompatibility distance as number of sequences, where A and B order is different from the majority case. A distance defined in this way can be effectively used to evaluate the possibility that two seeds together represent a possible homology signal. This distance is used to construct a maximum non-conflicting set of seeds in the following procedure.

A constant number of T best seeds is selected (T is 600 in current MISHIMA, because of memory limitations). Then the matrix of size TxT is constructed. Each cell of the matrix contains the incompatibility distance between two seeds. In the first step all of the selected T seeds are included and the matrix contains TxT distance values. Then the matrix is analyzed to find the sum of all numbers for every row. This sum corresponds to the total amount of incompatibility introduced by one seed. Now the seed which introduces the largest amount of incompatibility is removed from the set. The matrix is recalculated and the procedure iterates until no incompatibility is left unresolved. Finally only compatible seeds remain, which are used as alignment anchors. If all seeds are mutually incompatible with each other, there will be still one anchor left after removing the incompatibility. This anchor is used to divide the sequences into 2 parts, which are aligned separately.

The number of anchors found in all our example datasets is included in Additional file 1, in *.anchors files.

### Iterative procedure of dictionary analysis

After the dictionary step has finished and a set of non-conflicting seeds is selected, the algorithm will have a set of anchors, connecting the sequences. The sequences are then divided and regions between the anchors are aligned independently from each other. After all partial alignments are complete, they are concatenated to construct a final complete alignment.

Alignment of the regions between the seeds is performed using an external aligner provided the sequences are short enough. Otherwise MISHIMA is used again (recursively) to divide the sequences into shorter parts. The depth of the recursive operation in MISHIMA can be set with the command line option '-max-depth = x'. Since MISHIMA takes a constant time for each step of finding seeds and dividing the sequences, using a limited depth may improve the performance on some datasets.

**Figure 1:**
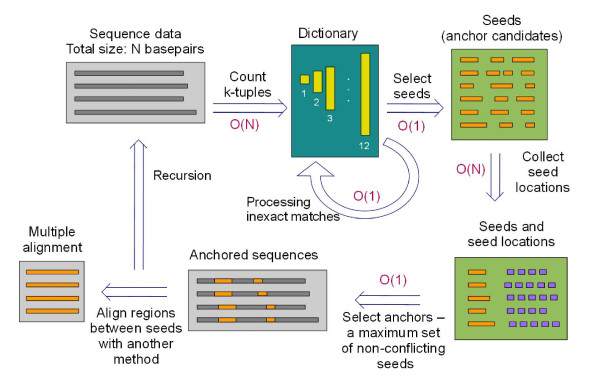
**Procedure of MISHIMA alignment and complexity of each step**.

The overall flow of MISHIMA algorithm is shown in Fig. 1.

### Assessing the alignment speed and quality

The performance of MISHIMA was evaluated on several example dataset, detailed in the Results section. We used several popular aligners for comparison: CLUSTAL W, MUSCLE, MAFFT and MLAGAN. The details, including version and parameters we used with these methods, are included in Additional file 1.

For assessing the alignment quality we used the sum-of-pairs alignment score [26], based on the pairwise sequence identity. The score is computed as arithmetic mean of pairwise identity scores of all sequence pairs in the alignment. Pairwise identity score is computed as following: S_ij _= 100*M_ij_/L, where M_ij _is the number of alignment positions where sequences i and j have the same nucleotide (not including gaps), and L is the length of complete multiple alignment (including gaps).

While a simulated dataset is necessary for a serious discussion of alignment quality, we believe that using the sum-of-pairs score is sufficient for evaluating MISHIMA's performance, for the following reasons: 1. Our main focus is on comparing MISHIMA+external aligner combination to external aligner alone. 2. We are mainly interested in verifying whether MISHIMA would not produce particularly big misalignments; the simplified score is sufficient for this purpose. 3. MISHIMA is targeting rather closely related sequences, which means that the correct alignments produced by almost any method will have about the same score, and misalignments will result in a noticeable score decrease.

The sum-of-pairs identity score was computed for all example alignments produced by MISHIMA and other methods.

## Results and Discussion

### Human mtDNA genomes

Complete human mitochondrial DNA (mtDNA) genome sequences were used as an example of very closely related sequences. As of July 16, 2009, a total of 4,718 complete human mtDNA genome sequences are available in DDBJ/EMBL/GenBank International Nucleotide Sequence Database. MISHIMA was able to successfully align hundreds of such genomes. Computation results can be seen in Additional file 1.

**Figure 2:**
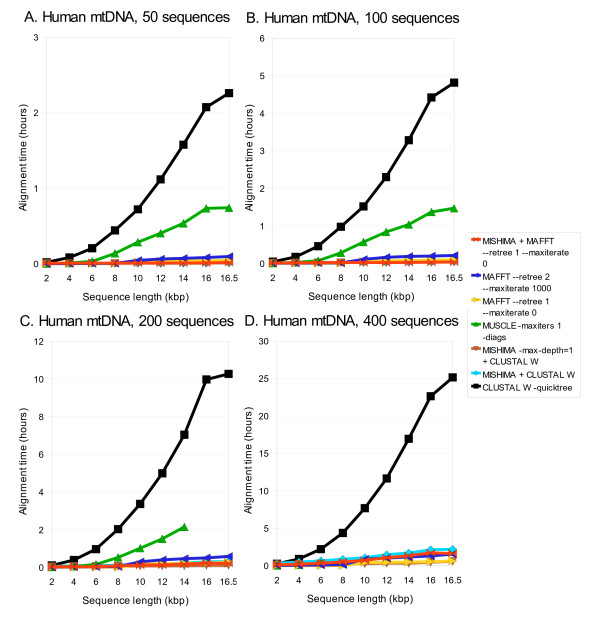
**Computation time of MISHIMA, CLUSTAL W, MUSCLE and MAFFT, on human mtDNA datasets of 50, 100, 200 and 400 sequences, complete and partial**. MUSCLE results are shown for all cases where it could complete the alignment.

We measured the time MISHIMA takes for aligning the whole and partial sequences and compared it with three popular aligners: CLUSTAL W, MUSCLE and MAFFT (Fig. 2 and Table 1). Partial sequences (with 2 kb increment) and complete ~16.5 kb sequences were used to see the effect of sequence length on computation time. A fixed length fragment was cut from the beginning of each sequence to produce partial sequences. Note that MISHIMA was using an external aligner (CLUSTAL W or MAFFT) to align the regions between the anchors, and their running time is counted in the MISHIMA total running time. 50, 100, 200 and 400 sequences were compared separately in order to examine effect of number of sequences on computation time. Nucleotide diversity for these datasets is within the range of 0.002-0.003.

**Table 1:** Alignment time comparison of various numbers of human mtDNA genomes (complete sequences)

	No. of sequences compared			
	
Method	50	100	200	400
MISHIMA + MAFFT	00:01:05	00:02:23	00:12:59	01:38:09

MISHIMA + CLUSTAL W -max-depth = 1	00:00:43	00:02:06	00:06:05	00:34:19

CLUSTAL W	02:15:32	04:48:59	10:16:22	25:08:23

MUSCLE	00:44:29	01:28:12	02:08:44	-----

MAFFT	00:02:43	00:05:44	00:16:31	00:38:51

Nucleotide diversity	0.003	0.002	0.002	0.002

CLUSTAL W is the slowest among the compared aligners, even with [-quicktree] option, followed by MUSCLE (Fig. 2). Fig. 2 shows that computation time of MISHIMA and MAFFT increases much slower than that of CLUSTAL W and MUSCLE. CLUSTAL W took 90-100 times longer time for complete mtDNA sequence alignment than it took aligning 2 kb fragments for all four cases. MISHIMA alignment time increased only 10 times between 2 kb and full length datasets. MUSCLE was unable to complete the alignment of 200 and 400 complete sequences. We used two kinds of option sets for MAFFT; [--retree 1 --maxiterate 0] (shown in orange) and [--retree 2 --maxiterate 1000] (shown in blue). Both option sets showed much faster results than either MUSCLE or CLUSTAL W. MISHIMA is as fast as MAFFT set at faster option (shown in orange) either when CLUSTAL W was used (shown in light blue) or when MAFFT was used (shown in red) as the external alignment program when 50, 100, and 200 human mtDNA sequences were compared.

When 400 human mtDNA sequences are compared, MAFFT set at faster option is clearly faster than MISHIMA. However, if we add [-max-depth = 1] option to MISHIMA (shown in light blue), both aligners finish in similar time. Because human mtDNA sequences are quite similar with each other, good seeds are found in abundance in the first iteration of dictionary analysis, and average block length (length of sequences between two adjacent anchor seeds) is already short enough for CLUSTAL W usage. Therefore, we recommend using this option when aligning closely related sequences, such as sequences from same species.

**Figure 3:**
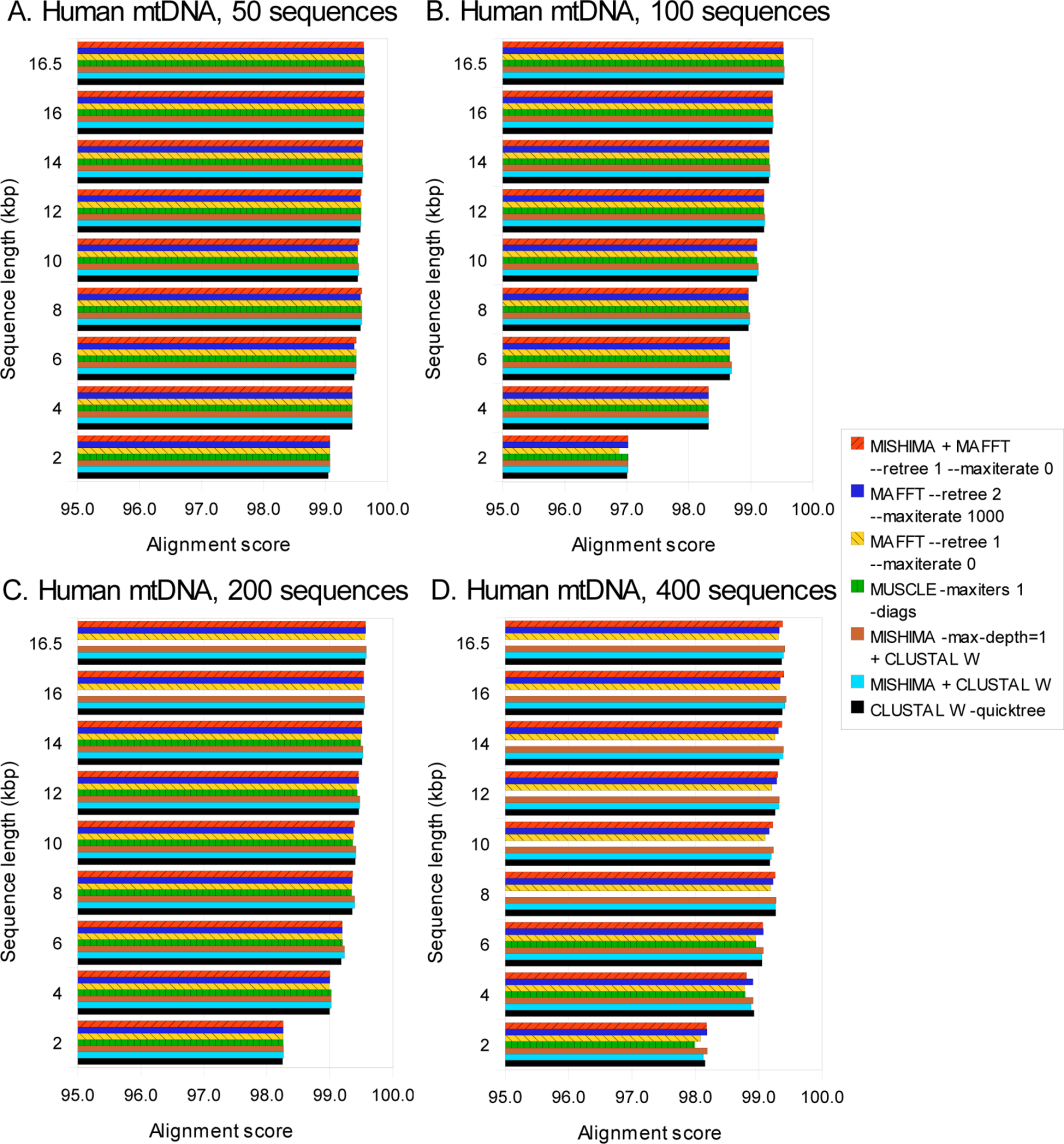
**Alignment score of alignments produced by MISHIMA, CLUSTAL W, MUSCLE and MAFFT, on human mtDNA datasets of 50, 100, 200 and 400 sequences, complete and partial**. MUSCLE results are shown for all cases where it could complete the alignment.

While the performance of MISHIMA is similar to that of MAFFT, it should be noted that only high similarity regions are aligned by MISHIMA, and the lower similarity parts are aligned with an external alignment program - either CLUSTAL W or MAFFT. This means that when CLUSTAL W is used as an external aligner, the alignment quality will be close to that of the CLUSTAL W complete alignment. Fig. 3 shows sum-of-pairs alignment score [26] of all alignments compared. MISHIMA alignment tends to have higher score than alignments produced by CLUSTAL W or MAFFT alone.

We also tried MLAGAN [24] on these datasets, but it did not work well, either producing corrupted alignment, or taking very long time (even compared to CLUSTAL W). Therefore MLAGAN results are not included in the figures.

### Mammalian mtDNA genomes

MISHIMA is powerful for aligning closely related nucleotide sequences, as shown in the above section. If more divergent sequences are compared, how does MISHIMA perform? We thus retrieved complete mtDNA genomes from various order of mammalian species. Average nucleotide difference for these datasets are within the range of 0.25-0.27.

**Figure 4:**
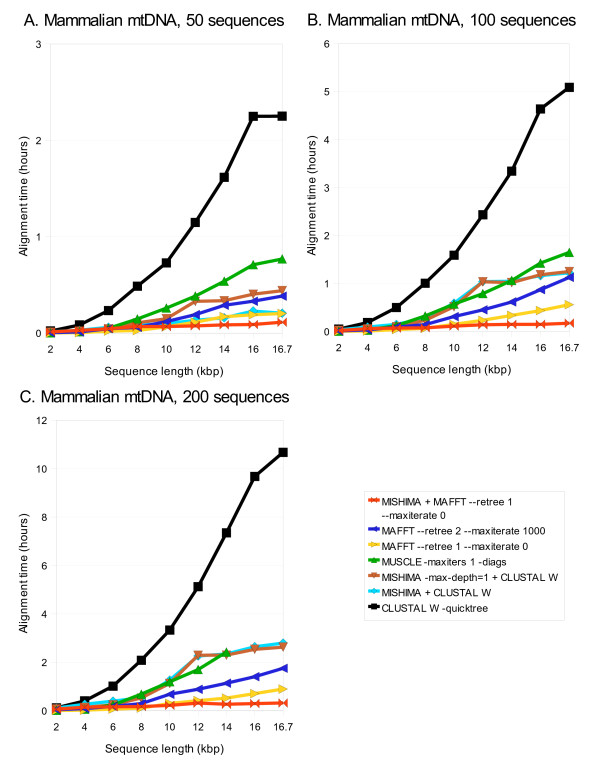
**Computation time of MISHIMA, CLUSTAL W, MUSCLE and MAFFT, on mammalian mtDNA datasets of 50, 100 and 200 sequences, complete and partial**. MUSCLE results are shown for all cases where it could complete the alignment.

Alignment times taken by MISHIMA (with various settings), CLUSTAL W, MUSCLE, and MAFFT for aligning datasets of 50, 100 and 200 complete mtDNA sequences are shown in Table 2. Scaling of the alignment time depending on sequence length is shown in Fig. 4: MISHIMA+MAFFT combination is the fastest, followed by MAFFT alone, MISHIMA+CLUSTAL W, MUSCLE. CLUSTAL W is much slower. MUSCLE could not complete the alignment of 200 complete mtDNA genomes. MLAGAN results are not shown, since it was not able to produce the alignment even for 2 kb fragments.

**Table 2:** Alignment time comparison of various numbers of mammalian mtDNA genomes (complete sequences)

	No. of sequences compared		
	
Method	50	100	200
MISHIMA + MAFFT	00:06:50	00:10:24	00:19:03

CLUSTAL W	02:15:04	05:05:20	10:40:31

MUSCLE	00:46:13	01:38:59	-----

MAFFT	00:12:13	00:33:36	00:53:38

Nucleotide diversity	0.249	0.274	0.262

If we compare computation times shown in Table 1 and Table 2, those of MISHIMA are certainly much slower for different mammalian species than for human individuals; 23 times and 13 times more for 50 and 100 sequences, respectively. This is because the number of good seeds for anchoring is reduced as sequence divergence increases. Computation results can be found in Additional file 1.

**Figure 5:**
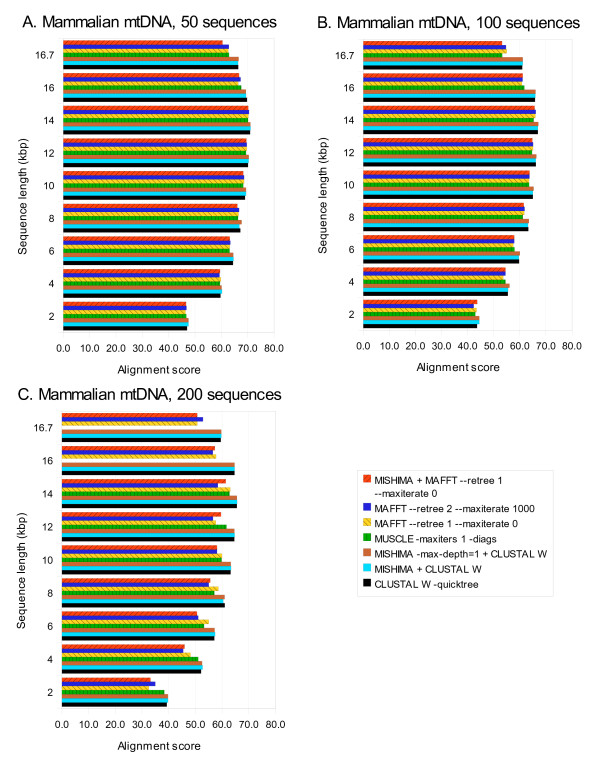
**Alignment score of alignments produced by MISHIMA, CLUSTAL W, MUSCLE and MAFFT, on mammalian mtDNA datasets of 50, 100 and 200 sequences, complete and partial**. MUSCLE results are shown for all cases where it could complete the alignment.

MISHIMA greatly accelerates the aligner that it is using: MISHIMA+CLUSTAL W is much faster than CLUSTAL W alone, MISHIMA+MAFFT is faster than MAFFT alone. Is this achieved by sacrificing the alignment quality? Fig. 5 shows the alignment score of all finished alignments. MISHIMA+CLUSTAL W score is about the same with the score of CLUSTAL W alone, also MISHIMA+MAFFT score is similar to score of MAFFT alone. Thus, the improvement in computation time is not achieved at the expense of alignment quality.

### Complete genomes of four different strains of bacteria

We are now moving from mtDNA genome sequences to much larger bacterial genomes. We first chose 4 strains of *Streptococcus pyogenes *as example. Each genome is about 2 MB, so the total dataset size is about 8 MB. CLUSTAL W could not produce the alignment even after 15 days, and computation was aborted. In good contrast, MISHIMA+CLUSTAL W could successfully produce the multiple alignment of these four genomes in less than three hours. In the course of alignment the dataset was separated into 485 segments, divided by 484 seeds. The average length of one segment was about 4 kb, which is much easier to align than complete sequences.

We used various values for [-max-depth = N] option: unlimited depth (default), 1, 2, and 3. Computation times for each case are shown in Table 3. Adding [-max-depth = 1] reduced the computation time in the case of 400 human mtDNA genome sequences (see Table 1), while activating this option for alignment of 4 genome sequences of *Streptococcus pyogenes *strains resulted in 3 times slower computation. As the number of iterations increased, computation time was reduced, and the computation time for the case of [-max-depth = 3] was slightly shorter than default unlimited depth (Table 3). With [-max-depth = 1] (number of recursive uses of MISHIMA anchoring algorithm is set to only 1), some sequence blocks may be quite long, and external aligner may take very long time aligning such blocks. Computation results can be seen in Additional file 1.

**Table 3:** Alignment time of 4 complete genomes of *Streptococcus pyogenes *with various settings of MISHIMA

Method	Alignment time
MISHIMA -max-depth = 1 -aligner = clustalw	10:08:52
MISHIMA -max-depth = 2 -aligner = clustalw	02:54:48
MISHIMA -max-depth = 3 -aligner = clustalw	02:33:44
MISHIMA (unlimited depth) -aligner = clustalw	02:43:03
MISHIMA -max-depth = 1 -align-seeds-only	00:00:27

In any case, it is clear that MISHIMA can be used to align multiple complete bacterial genomes, as long as they are closely related. The alignment of four strains of *Streptococcus pyogenes *revealed several large insertions and deletions, some more than 10 kb long. If one is interested in quickly finding such large deletions, we recommend use of [-align-seeds-only] option, so that the external aligner is not activated. In this way only seed anchoring is conducted, which is much faster (only 27 seconds in *S. pyogenes *example) than producing full multiple alignment.

### Complete genomes of six different strains of bacteria

**Figure 6:**
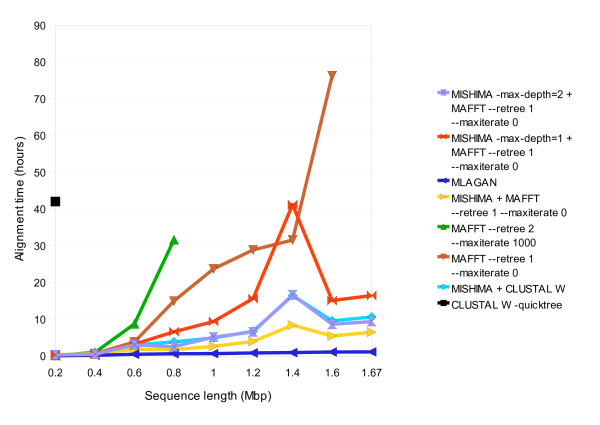
**Computation time of MISHIMA, CLUSTAL W, MAFFT and MLAGAN on *Helicobacter pylori *datasets of 6 sequences, complete and partial**.

**Figure 7:**
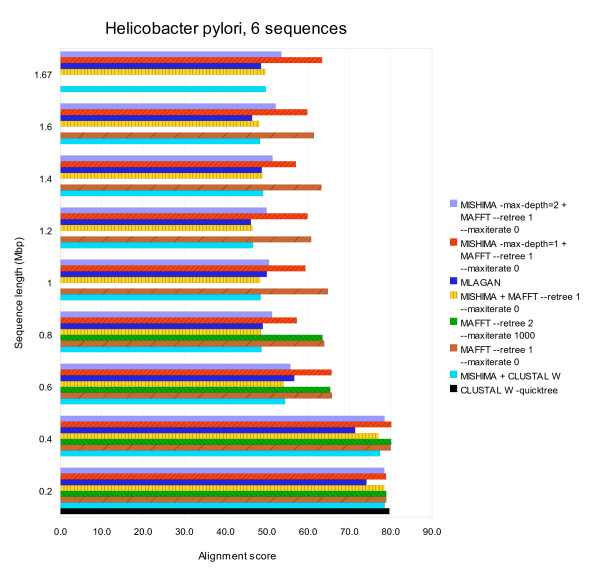
**Alignment score of alignments produced by MISHIMA, CLUSTAL W, MAFFT and MLAGAN on *Helicobacter pylori *datasets of 6 sequences, complete and partial**.

We then moved to a more complex dataset - 6 complete genomes of different strains of *Helicobacter pylori *- each about 1.7 MB. Average nucleotide difference of the dataset is 0.133. Neither CLUSTAL W, MAFFT nor MUSCLE were able to complete the alignment even after 3 days. Therefore we decided to also run tests on fragments of these genomes; we chopped 200 Kb from the beginning of every sequence, then 400 Kb, 600 Kb, and so on, until 1.6 Mb. The computation time of various methods can be seen in Fig. 6, and alignment score is shown in Fig. 7. MUSCLE was unable to align even the 200 Kb dataset, while CLUSTAL W took 42 hours. MAFFT managed it within 3 minutes - faster than MISHIMA which took 21 minutes with default settings. However, as longer fragments were attempted, MAFFT was slowing down rapidly. With 600 Kb fragments MISHIMA was 2 times faster than MAFFT at its fastest setting [--retree 1 --maxiterate 0]. At the 1.2 Mb length, MISHIMA was more than 6 times faster compared to MAFFT (Fig. 6). This time we could also use MLAGAN, which performed very fast in this dataset. The alignment score of MISHIMA is comparable to that of other methods (Fig. 7).

MISHIMA+MAFFT combination performed well on this dataset. Adding options [-max-depth = 1] and [-max-depth = 2] allowed to save time and improve the alignment score with this dataset.

### Larger datasets

**Figure 8:**
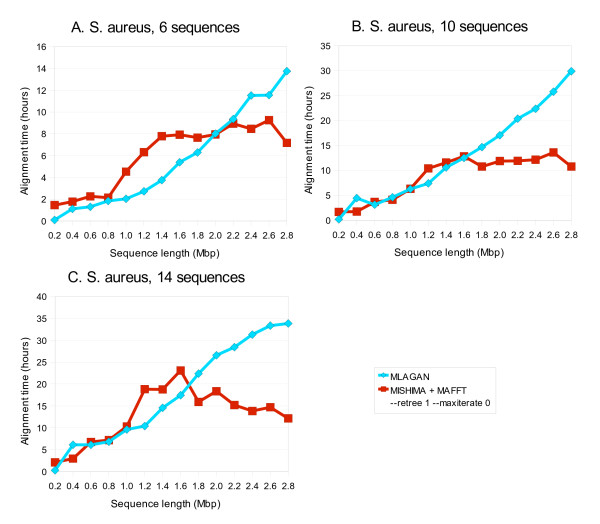
**Computation time of MISHIMA and MLAGAN on *Staphylococcus aureus *datasets of 6, 10 and 14 sequences**.

We tried MISHIMA on larger bacterial datasets. We used fragmented sequences of 14 genomes of *Staphylococcus aureus*. CLUSTAL W, MUSCLE and MAFFT were taking too long time to align this dataset. MLAGAN was still able to produce the alignment. MISHIMA was operating with the default options in this example, using MAFFT as an external aligner. MISHIMA and MLAGAN alignment times are comparable to each other with partial datasets. When increasing the alignment length, MISHIMA+MAFFT combination becomes faster (Fig. 8).

Interestingly, MISHIMA alignment time starts to decrease when the sequences are getting closer to complete genome. The reason is that more complete sequences allow for the construction of a set of anchors that leaves smaller unaligned sequence block in the end. This effect is particularly apparent with 14 sequences (Fig. 8).

**Figure 9:**
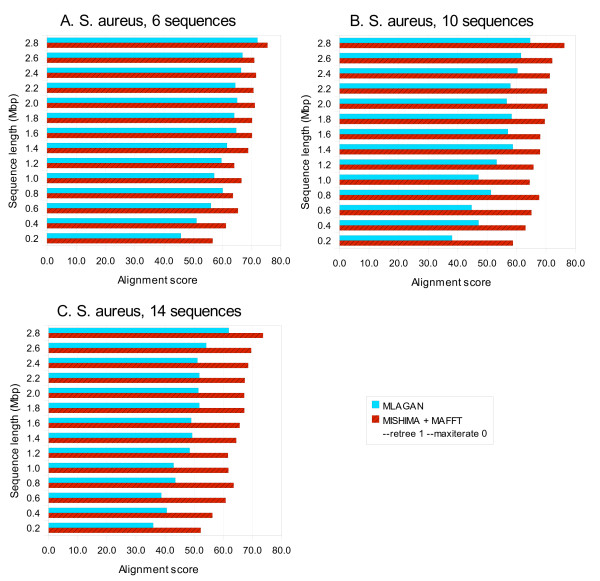
**Alignment score of alignments produced by MISHIMA and MLAGAN on *Staphylococcus aureus *datasets of 6, 10 and 14 sequences**.

The alignment score does not show any big problems with MISHIMA alignment (it is usually higher than that of MLAGAN alignment, Fig. 9). So we can conclude that MISHIMA does not sacrifice the alignment quality for fast computation.

At present, we were not able to compare the time and score of MISHIMA+MAFFT with just MAFFT, however from previous examples we have seen that MISHIMA+MAFFT alignment is similar in quality to MAFFT alignment. Thus by using MISHIMA we can produce MAFFT-quality alignment even for large datasets where we can't use MAFFT alone.

This example shows that MISHIMA is effective on large datasets, including those that are very difficult to align with other programs.

### Random sequences

Multiple alignments should be conducted on homologous sequences. However, there will always be a possibility of non-homologous sequences being erroneously included for multiple alignment. It is therefore ideal to detect such non-alignable sequences instantaneously and MISHIMA can do just that. We demonstrate MISHIMA's ability to detect unalignable sequences by using random sequences. Two sets of random sequences (50 and 100 sequences of 20 kb length) were generated using newly built random sequence generator (K. Kryukov, unpublished). We used MISHIMA with [-align-seeds-only -max-depth = 1] options. As expected, MISHIMA could not detect any seeds, which is a good indication that the data is very hard or impossible to align. Quick examination of the result confirmed that the sequences could not be aligned. MISHIMA took only 13 and 15 seconds to produce the results for 50 and 100 sequences, while CLUSTAL W took hours trying to align these random and non-homologous sequences (Table 4).

**Table 4:** Alignment time of 20 kb random sequences

Number of sequences	CLUSTAL W	MISHIMA
50	03:55:05	00:00:13

100	08:43:09	00:00:15

We therefore recommend to first try using MISHIMA with [-align-seeds-only -max-depth = 1] options on new data. By this way you will be able to quickly know if there are any problems with the sequences. If MISHIMA was able to find seeds and produce a rough alignment, then the sequences are fine and normal MISHIMA alignment procedure can be used. In fact, computation times using this option set for human complete mtDNA genomes are 00:00:18, 00:01:15, and 00:14:38 for 50, 100, and 200 sequences, respectively. When [-max-depth = 1] option is also activated, computation times for 200 and 400 sequences are 00:00:51 and 00:02:27, respectively. These are much shorter than MISHIMA default for producing full multiple alignments.

## Conclusion

We developed a heuristic method for multiple sequence alignment that can greatly speed up alignment of large datasets - those consisting of hundreds of sequences, as well as those with very long sequences, such as complete bacteria genomes.

MISHIMA depends on either MAFFT (default option), or CLUSTAL W (with [-aligner = clustalw]) for aligning the areas between the "seeds" to produce the complete alignment. However even without other programs MISHIMA can be used to quickly produce a rough alignment where only seeds are aligned. This can be very useful for quick evaluation of a new dataset before applying a more thorough alignment procedure.

Given the rapidly increasing number of sequenced organisms, there is a growing interest in comparing closely related species. MISHIMA is particularly helpful for such analysis due to its ability to quickly locate homology signals.

## Availability and requirements

Project name: MISHIMA

Project home page: http://esper.lab.nig.ac.jp/study/mishima/

Operating system: Windows

Programming language: C (not open source).

Other requirements: For using with CLUSTAL W, CLUSTAL W version 2.0.10 or later should be installed. For using with MAFFT, MAFFT version 6.707b or later should be installed.

License: Binary is free for any use.

Any restrictions to use by non-academics: None.

## Authors' contributions

KK came up with the idea of heuristic homology search, developed the software, ran tests and drafted the manuscript. NS contributed to the design of the algorithm, did additional testing on large datasets, and improved the manuscript. Both authors read and approved the final manuscript.

## Supplementary Material

Additional file 1 Index of/study/mishima/supplementary-data Click here for file
