# Supplementary material for: MISHIMA - a new method for high speed multiple alignment of nucleotide sequences of bacterial genome scale data
Source: BMC Bioinformatics. 2010 Mar 18;11:142. doi: 10.1186/1471-2105-11-142 (PMC2848238; doi:10.1186/1471-2105-11-142)
Supplement: Additional file 1 — Index of/study/mishima/supplementary-data [file 1471-2105-11-142-S1.DOCX]

**All original sequences and multiply aligned sequences are availabe at**

[**http://esper.lab.nig.ac.jp/study/mishima/supplementary-data/**](http://esper.lab.nig.ac.jp/study/mishima/supplementary-data/)

**Index of /study/mishima/supplementary-data**

- [Parent Directory](http://esper.lab.nig.ac.jp/study/mishima/)
- [MISHIMA-Benchmark-01-Human-mtDNA.zip](http://esper.lab.nig.ac.jp/study/mishima/supplementary-data/MISHIMA-Benchmark-01-Human-mtDNA.zip)
- [MISHIMA-Benchmark-02-Mammalian-mtDNA.zip](http://esper.lab.nig.ac.jp/study/mishima/supplementary-data/MISHIMA-Benchmark-02-Mammalian-mtDNA.zip)
- [MISHIMA-Benchmark-03-S-pyogenes-x4.zip](http://esper.lab.nig.ac.jp/study/mishima/supplementary-data/MISHIMA-Benchmark-03-S-pyogenes-x4.zip)
- [MISHIMA-Benchmark-04-H-pylori-x6.zip](http://esper.lab.nig.ac.jp/study/mishima/supplementary-data/MISHIMA-Benchmark-04-H-pylori-x6.zip)
- [MISHIMA-Benchmark-05.01-S-aureus-x6.zip](http://esper.lab.nig.ac.jp/study/mishima/supplementary-data/MISHIMA-Benchmark-05.01-S-aureus-x6.zip)
- [MISHIMA-Benchmark-05.02-S-aureus-x10.zip](http://esper.lab.nig.ac.jp/study/mishima/supplementary-data/MISHIMA-Benchmark-05.02-S-aureus-x10.zip)
- [MISHIMA-Benchmark-05.03-S-aureus-x14.zip](http://esper.lab.nig.ac.jp/study/mishima/supplementary-data/MISHIMA-Benchmark-05.03-S-aureus-x14.zip)
- [MISHIMA-Benchmark-06-Random-sequence.zip](http://esper.lab.nig.ac.jp/study/mishima/supplementary-data/MISHIMA-Benchmark-06-Random-sequence.zip)
- [MISHIMA-Benchmark-Scripts.zip](http://esper.lab.nig.ac.jp/study/mishima/supplementary-data/MISHIMA-Benchmark-Scripts.zip)
